# GENTRIFICATION AND COGNITIVE DECLINE IN OLDER ADULTS: MULTI-ETHNIC STUDY OF ATHEROSCLEROSIS

**DOI:** 10.1093/geroni/igae098.4200

**Published:** 2024-12-31

**Authors:** Jana Hirsch, Kari Moore, Reyhaneh Akbar Nejad Yazdi, Lilah Besser, Timothy Hughes, Yvonne Michael

**Affiliations:** Drexel University Dornsife School of Public Health, Philadelphia, Pennsylvania, United States; Drexel University Dornsife School of Public Health, Philadelphia, Pennsylvania, United States; Drexel University Dornsife School of Public Health, Philadelphia, Pennsylvania, United States; University of Miami, Boca Raton, Florida, United States; Wake Forest University School of Medicine, Winston-Salem, North Carolina, United States; Drexel University Dornsife School of Public Health, Philadelphia, Pennsylvania, United States

## Abstract

Gentrification, transition of neighborhoods from a state of disinvestment to reinvestment, introduces new amenities potentially beneficial for health of residents who are able to remain. However, the displacement of existing residents may result in stress and diminished social connection. These disparate influences of gentrification may positively or negatively influence older adults’ brain health. We used a sample of 1,292 participants from the Multi-Ethnic Study of Atherosclerosis (MESA) to examine associations between gentrification from 2000 to 2010 and cognitive function change between Exams 5 (2010-2012) and 6 (2016-2018). Neighborhoods were categorized as continually advantaged (ineligible to gentrify), continually disadvantaged (eligible to gentrify but not gentrified), and gentrified. We categorized three continuous cognitive measures as maintained/improved score versus decline in score from Exam 5 to 6: CASI (version 2), global cognitive function; Digit Symbol Coding (DSC) task, processing speed; and Digit Span (DS), short term memory. Compared to those living in continually disadvantaged neighborhoods, participants residing in gentrified neighborhoods had 5% lower risk of CASI decline and 2% lower risk of DSC decline. More pronounced, those residing in continually advantaged neighborhoods had 10% lower risk of CASI decline, 6% lower risk of DS decline, and 8% lower risk of DSC decline. After adjustment for age, gender, race/ethnicity, language of administration, education, income, working status, and APOE4, only DSC declines remained statistically significant. Among a multi-ethnic and geographically diverse population, we observed a linear relationship through which risk of cognitive decline decreased with higher level and improvement of neighborhood socioeconomic conditions.

